# Supplementary material for: Comparison of the Relationship between Visual Acuity and Motor Function in Non-Elderly and Elderly Adults
Source: J Clin Med. 2023 Mar 3;12(5):2008. doi: 10.3390/jcm12052008 (PMC10003822; doi:10.3390/jcm12052008)
Supplement: Supplementary file 1 [file jcm-12-02008-s001.zip › jcm-2197155-supplementary.pdf]

Supplementary Material

Table S1. The comparison of each parameter between the N and L groups in non-elderly male participants.

Non-elderly men	Total (n = 45)	N (n = 35)	L (n = 10)	P value
Age (yrs)	57 ± 5.8	56.2 ± 6.1	59.9 ± 3.5	0.085
BMI (kg/m²)	24.7 ± 3.1	24.5 ± 3.2	25.5 ± 2.5	0.423
BFP (%)	24.4 ± 4.2	23.8 ± 4.3	26.4 ± 3.1	0.088
SMI (kg/m²)	7.9 ± 0.6	7.9 ± 0.5	7.8 ± 0.7	0.813
Back muscle strength (kg)	119.5 ± 25.3	124.1 ± 24.7	105.5 ± 22.6	0.042*
Gait speed (m/s)	2.2 ± 0.3	2.2 ± 0.3	2.2 ± 0.2	0.617
Length of COP sway (eyes open) (cm/s)	1.7 ± 0.3	1.6 ± 0.3	1.9 ± 0.3	0.086
Length of COP sway (eyes closed) (cm/s)	2.4 ± 0.7	2.3 ± 0.7	2.6 ± 1	0.228
Surrounding area of COP sway (eyes open) (cm²)	2.9 ± 1.2	2.8 ± 1.2	3.1 ± 0.9	0.503
Surrounding area of COP sway (eyes closed) (cm²)	3.9 ± 2.2	3.6 ± 1.9	4.9 ± 3.1	0.121

Visual acuity(N/L), normal visual acuity group (N group)/low visual acuity group (L group); BMI, body mass index; BFP, body fat percentage; SMI, skeletal muscle mass index, COP, center of pressure. *P < 0.05.

Table S2. The comparison of each parameter between the N and L groups in non-elderly female participants.

Non-elderly women	Total (n = 95)	N (n = 70)	L (n = 25)	P value
Age (yrs)	54.9 ± 6.9	54.8 ± 7	54.9 ± 6.7	0.964
BMI (kg/m²)	23.3 ± 4	23.1 ± 3.9	23.8 ± 4.4	0.469
BFP (%)	33.4 ± 7.1	33.1 ± 7	34 ± 7.5	0.583
SMI (kg/m²)	6.2 ± 0.7	6.2 ± 0.6	6.3 ± 0.7	0.419
Back muscle strength (kg)	61.2 ± 15.7	63.9 ± 15.6	53.4 ± 13.5	0.009*
Gait speed (m/s)	2 ± 0.3	2.1 ± 0.3	1.9 ± 0.2	0.084
Length of COP sway (eyes open) (cm/s)	1.3 ± 0.3	1.3 ± 0.4	1.3 ± 0.3	0.678
Length of COP sway (eyes closed) (cm/s)	1.5 ± 0.6	1.5 ± 0.6	1.5 ± 0.4	0.909
Surrounding area of COP sway (eyes open) (cm²)	2.2 ± 1.4	2.1 ± 1.3	2.7 ± 1.5	0.056
Surrounding area of COP sway (eyes closed) (cm²)	2.4 ± 2.1	2.5 ± 2.3	2.4 ± 1.2	0.824

Visual acuity(N/L), normal visual acuity group (N group)/low visual acuity group (L group); BMI, body mass index; BFP, body fat percentage; SMI, skeletal muscle mass index, COP, center of pressure. *P < 0.05.

Table S3. The comparison of each parameter between the N and L groups in elderly male participants.

elderly men	total(n=72)	N(n=48)	L(n=24)	P value
age(yrs)	71.8±4.8	70.8±3.9	73.8±5.8	0.012*
BMI(kg/m2)	24±2.8	23.7±2.7	24.6±3	0.202
BFP (%)	23±4	22.7±3.8	23.5±4.4	0.419
ASMI(kg/m2)	7.6±0.7	7.6±0.7	7.6±0.8	0.441
Back muscle strength(kg)	106.7±24	106.7±23.6	106.6±25.4	0.963
Gait speed(m/s)	2.1±0.3	2.1±0.3	2.0±0.4	0.024*
Length of COP sway(eyes open)(cm/s)	1.9±0.7	1.8±0.6	2±0.8	0.334
Length of COP sway(eyes closed)(cm/s)	2.5±1.2	2.5±1.1	2.6±1.4	0.708

Surrounding area of COP sway (eyes open)(㎠)	2.9±1.7	2.8±1.7	3.1±1.7	0.379
Surrounding area of COP sway (eyes closed)(㎠)	4±2.7	3.7±2.8	4.4±2.5	0.990

Visual acuity(N/L), normal visual acuity group (N group)/low visual acuity group (L group); BMI, body mass index; BFP, body fat percentage; SMI, skeletal muscle mass index, COP, center of pressure. *P < 0.05.

Table S4. The comparison of each parameter between the N and L groups in elderly female participants.

elderly women	total(n=83)	N(n=54)	L(n=29)	P value
age(yrs)	70.3±5.1	69.9±4.4	71.1±6.3	0.305
BMI(kg/m2)	23.1±3.8	22.7±3.7	23.8±3.9	0.203
BFP (%)	32.3±6.8	31.5±6.5	33.8±7.3	0.163
ASMI(kg/m2)	6.1±0.7	6.1±0.7	6±0.6	0.675
Back muscle strength(kg)	58.5±17.6	57.5±16.8	60.4±19.3	0.514
Gait speed(m/s)	1.8±0.2	1.9±0.2	1.7±0.2	0.009*
Length of COP sway(eyes open)(cm/s)	1.6±0.4	1.6±0.4	1.5±0.4	0.486
Length of COP sway(eyes closed)(cm/s)	1.9±0.6	1.9±0.6	1.8±0.6	0.770
Surrounding area of COP sway (eyes open)(㎠)	2.5±1.4	2.6±1.3	2.5±1.8	0.919
Surrounding area of COP sway (eyes closed)(㎠)	2.9±2.2	2.7±1.5	3.2±3.1	0.328

Visual acuity(N/L), normal visual acuity group (N group)/low visual acuity group (L group); BMI, body mass index; BFP, body fat percentage; SMI, skeletal muscle mass index, COP, center of pressure. *P < 0.05.
